# Psychometric Properties of the Service Leadership Attitude Scale in Hong Kong

**DOI:** 10.3389/fpsyg.2019.01070

**Published:** 2019-05-10

**Authors:** Daniel T. L. Shek, Wen Yu Chai

**Affiliations:** Department of Applied Social Sciences, The Hong Kong Polytechnic University, Hong Kong, China

**Keywords:** assessment, leadership, service leadership, scale validation, Hong Kong

## Abstract

Positive attitude to service leadership is fundamental for the development of successful leadership qualities in the service-dominated economy. The purpose of this study was to examine the psychometric properties of the 46-item Service Leadership Attitude Scale (SLA) in Hong Kong. A total of 2,240 undergraduate students in Hong Kong (mean age = 20.44±1.64 years; 66.9% were female) completed a questionnaire containing the 46-item SLA and other leadership-related scales. The psychometric properties of SLA were assessed using confirmatory factor analyses (CFA), reliability analyses, measurement invariance tests, and convergent validity test. Previous exploratory factor analyses suggested a seven-factor model of SLA which was supported by CFA in this study. By adding an additional dimension (“Implicit leadership theory”) in the scale, the final eight-factor model with 46 items showed superior fit using CFA. Factorial invariance tests showed that the factor structure was invariant in terms of construct dimensionality, factor loadings, intercepts, and factor covariance across gender and subgroups split based on “odd” and “even” case numbers. Convergent validity tests showed that the scale scores were correlated with several leadership-related scales. The scale and subscales also demonstrated good internal consistency reliability. This study is the first scientific work to validate a measure of attitude to service leadership via CFA. It contributes significantly to the development and validation of measures of attitude to service leadership, particularly in the higher education sector.

## Introduction

During the past decades, our economic and social structures have changed a lot because of the transformation of economic mode, rapid economic globalization, and technological advancement. One dramatic change has been the growing importance of “service” in economic development and the global predominance of service-based economy, in which unique and good quality of service provided to customers has become the “competitive advantage” of organizations which cannot be easily copied (Gronfeldt and Strother, 2006; Barrett and Davidson, 2008). For example, up to the turn of the century, service economy had contributed more than 70% of the GDP in OECD countries, while manufacturing economy contributed only 20% of the GDP (Organization for Economic Co-operation and Development (OECD), 2000). Similarly, in the United States, service sectors contributed 77% of the total national GDP in 2017, while manufacturing sectors only contributed 12% (World Bank, 2017). With specific reference to Hong Kong, according to the statistics provided by the Census and Statistics Department of The Government of HKSAR (2018), the service sectors had contributed 92.9–92.4% of the total GDP of Hong Kong’s economic development from 2013 to 2017. In the global world, the impact of service-dominated economy has been intensified by economic globalization and rapid-advancement of science and technology, which significantly reshapes and transforms organizational structures and behaviors and calls for the emergence of new leadership concepts and paradigms (e.g., Sartori et al., 2018).

Several major transformations and developments of leadership paradigms could be identified in the global service-based economy (Shek et al., 2015a,b). Firstly, in the predominance of service-based economy and economic globalization, division of labor is more fluid and complex; the organizational structure becomes flatter, more organic, and decentralized, which all challenge the conventional concept of leadership that leadership is merely held by one or few individuals in the higher or authority positions (Gronn, 2002; Houghton et al., 2002; Bolden, 2011). Under these economic and organizational transitions, leadership and organizational success is no longer determined by one or few persons, but by team members’ collective capacities to influence others in completing given tasks (Pearce and Conger, 2002). Therefore, distribution, decentralization, and sharing of leadership authority are important for organizations to act in a flexible and responsive manner in service-oriented markets (Houghton et al., 2002). Therefore, during the past decades, there has been the emergence of concepts of “distributed leadership” and “shared leadership.” Both stress that leadership is a dynamic and interactive influencing process among members of a team or organization for achieving organizational goals (Bligh et al., 2006). These leadership concepts also stress that “many people will have the potential to exercise leadership in any organization” (Harris, 2008, p. 173–174).

Secondly, under service-based economy, fast-changing economic situation, and economic globalization, leaders should possess not only intellectual competences such as problem-solving and strong decision-making skills but also other competences such as emotional and spiritual intelligence. In service-based economy, employees’ initiative, creativity, and shared-responsibility also become indispensable (Gronfeldt and Strother, 2006). To motivate employees, leaders’ understanding of employees’ emotions and how their own emotions would influence employees are very important (George, 2000). Meanwhile, leaders’ spiritual intelligence has also been identified as an important competence to shape employees’ intrinsic motivation to work. It can be reasoned that spiritual leaders could get in touch with the core values of their employees, create values and visions congruent among their team members, and shape the spiritual survival of their followers, which ultimately contributes to the organizational commitment and success (Fry, 2003).

Thirdly, the ethical dimension of leadership has gained more attention in recent years. Moral character attributes such as integrity, fairness, trustworthiness, and honesty have been highlighted as indispensable for leadership effectiveness (Brown and Treviño, 2006). Ethical leaders could establish and maintain positive relationships with different stakeholders such as employees and customers through providing safe and healthy working environment and safe products (Zhu et al., 2014), which is vital for long-term organizational success in service-based economy. On the contrary, unethical leaders could ruin the trust of their employees, stakeholders, and customers, which finally leads to the failure of the organization (Sama and Shoaf, 2007; Shek et al., 2019).

The last but not the least, the economic and organizational changes call for the importance of self-leadership in leaders themselves and in the whole organization. Self-leadership refers to “an individual level process perspective through which men and women influence themselves to control their own actions and thinking” (D’Intino et al., 2007, p. 105) in order to achieve higher level of performance and success (Houghton et al., 2002). It involves processes of self-knowledge, self-awareness, and self-reflection that serve as the basis for “leading from within” (Dhiman, 2018, p. 21). The element of self-leadership is actually stressed in several contemporary leadership concepts such as spiritual leadership, authentic leadership, and servant leadership. In a fast-changing and volatile economic environment, self-leadership is a very important quality for leaders to deal with stress, challenges, and even failures encountered in leading organizations (D’Intino et al., 2007). Meanwhile, to promote self-leadership among employees and followers is also important for organizational and team empowerment (Houghton et al., 2002).

To capture and embrace leadership changes, characteristics, and requirements in service economy, a new concept “service leadership” has emerged and been proposed by scholars in recent years. Some scholars regarded service leadership as a set of customer- or service-centered competences important in service sectors such as business-oriented competences, relationship-oriented competences, and self-oriented competences (Testa and Sipe, 2012). O’Malley (2005, p. 12) talked about service leadership as “germane to providing differentiating service from the inside out” and proposed that service leadership was not just the responsibility of leaders and managers but everyone should be accountable for it. Gronfeldt and Strother (2006) defined service leadership to be a “culture” or “collective mind-set” that “empowers the organization to strategize its promises, design its processes, and engage its people in a proactive quest for competitive advantage” (p. 7). This stresses that leadership is not the privilege of people at top positions, but exists in every employee’s beliefs and behaviors as one integral part of his or her job duties (Heifetz and Linsky, 2006). Testa (2004) argued that leadership style in service-dominated economy has its own characteristics, including the emphasis on “reciprocity,” good communication, and trust-building between leaders and employees. This helps promote and maintain positive perceptions and attitudes of employees which are vital for employees to provide quality services. Snell et al. (2015a) argued that service leadership was “the distributed practice of people-oriented leadership for service” (p. 375). They proposed three assumptions of service leadership. First, service leadership requires distributed leadership that everyone takes up leadership roles; it is effective only when it exists in each member’s responsibility. Second, establishing and maintaining positive social relationship between service provider and service recipient is vital for successful service leadership. Third, effective service leadership embraces a spectrum of attributes such as good character and respecting others, and abilities such as active listening and effective communication.

In view of the important role of service economy in Hong Kong and other parts of the world, the Hong Kong Institute of Service Leadership and Management (HKI-SLAM) proposed a framework of service leadership as a new conceptualization of leadership and new expected manpower-development requirement in Hong Kong. According to Chung (2011), one of the key founders of HKI-SLAM, service leadership is defined as “satisfying needs by consistently providing quality personal service to everyone one comes into contact with, including one’s self, others, groups, communities, systems, and environments.” By considering challenges of service-economy to leadership requirements, HKI-SLAM proposed three major components of effective service leadership: competences, character, and care (Shek and Lin, 2015). Specifically, an effective service leader should possess multiple competences including intrapersonal competences (e.g., emotional management) and interpersonal competences (e.g., positive interpersonal relationships). Meanwhile, effective service leaders should possess moral character and disposition of care to make ethical judgment and actions and to act on behalf of wellbeing of themselves, their employees, and the community.

Furthermore, seven core beliefs about service leadership are intrinsic to the HKI-SLAM model (Shek and Lin, 2015). First, service leadership is regarded as service to satisfy the needs of oneself, other people, and the whole community in an ethical way. Second, it is proposed that every person has the potential to execute and improve his or her leadership every day. Third, service leadership effectiveness is determined by three things: domain-specific task competences, moral character, and caring disposition. Fourth, besides serving others, service leaders should also serve themselves to improve their own competences and skills. Fifth, service leaders should consistently provide high-quality service to anyone they encounter with including themselves. Sixth, service leadership paradigm has long existed as an important paradigm in the history of human society. Seventh, it is maintained that individuals with competence, character, and care could gain higher-paid and higher-status positions. The HKI-SLAM framework of service leadership is innovative and distinct from contemporary leadership theories such as transformational leadership and servant leadership. For example, while servant leadership emphasizes serving others and transformational leadership stresses transcending self-interest for the organizational benefits, service leadership expands the scope of service to include serving oneself, others, and the system (Shek and Lin, 2015).

Although service leadership is mainly stressed in organization and workplace, how to nurture its related knowledge, attitudes, and behaviors among young people such as university students has important reference for how universities may prepare young people as leaders. In Hong Kong, with the transformation from a manufacturing-based to a service-based economy and handover to China in 1997, how to prepare young people to be effective service leaders is top on the agenda of the society (Jaffee, 2012; Shek et al., 2013). Along with the transformation of Hong Kong’s 3-year structure of undergraduate education to a 4-year one, students’ generic knowledge and skills have been stressed and leadership development initiatives and programs have been incorporated in the new curriculum. To promote service leadership education among university students in Hong Kong, HKI-SLAM collaborated with the Victor and William Fung Foundation and the eight public universities in Hong Kong funded by the University Grants Committee (UGC) to launch the Li and Fung Service Leadership Initiative in 2012 (HKI-SLAM, 2019). Funded by a HK$40 million grant, different universities either strengthened their existing subjects to promote service leadership or developed new service leadership programs based on HKI-SLAM framework and core beliefs of service leadership. For example, Lingnan University strengthened their Service-Learning projects to nurture their students’ service leadership knowledge and attitudes (Snell et al., 2015b). City University of Hong Kong integrated their service leadership education into their broad entrepreneurship and social innovation program (Wong and Chandra, 2015). At The Hong Kong Polytechnic University, a credit-bearing subject of service leadership was developed to nurture undergraduate students’ service leadership knowledge, attitudes, and behaviors in the new 4-year undergraduate program (e.g., Shek et al., 2013; Shek and Lin, 2014).

Although the concept of service leadership has been rigorously promoted and related programs were developed in higher education institutions in Hong Kong, a major limitation is that no validated measures have been developed to assess students’ service leadership knowledge, attitude, and behavior. Particularly, no validated measures have been developed to assess attitude to service leadership. Attitude generally refers to a psychological tendency that an individual evaluates an object or entity in terms of degree of favor or disfavor, liking or disliking, and good or bad (Eagly and Chaiken, 1993; Ajzen and Fishbein, 2000). Attitude is important to service leadership competence since “competence is generally defined as consisting of integrated pieces of knowledge, skills, and attitudes” (Baartman and de Bruijn, 2011, p. 126). Also, attitude is important because it is the underpinning of behaviors (Reid, 2011) and it influences behavior through behavioral intention (Anker et al., 2010). As claimed by Reid (2011, p. 6), “Attitudes serve a vital purpose. The attitudes held by an individual help that person to make sense of the world around, sense of themselves and sense of relationship.” Therefore, it is important to measure attitude to service leadership.

To assess people’s attitude to service leadership in Hong Kong, a set of measures were developed by researchers from the eight UGC-funded higher education institutions in Hong Kong with The Hong Kong Polytechnic University being the lead institution. The original version was the Long-Form Service Leadership Attitude Scale (SLA-LF-132) which contains 132 items under eight domains. These include “Service orientation,” “Belief that everyone can be a leader,” “Distributed leadership,” “Employability,” “Personalized service,” “Attitudes toward service,” “E = CCC (i.e., effective service leadership is composed by character, competences, and caring disposition),” and “Commitment to continuous improvement” (Shek et al., 2017). The eight domains and the 132 items were generated based on HKI-SLAM framework of service leadership and extensive literature review on service leadership, related leadership concepts, and leadership characteristics stressed in service-based economy, such as distributed leadership, spiritual leadership, moral leadership, and self-leadership (Shek et al., 2017; Ma et al., 2018). The detailed theoretical and conceptual framework of SLA and its relationship with other leadership concepts can be seen in Ma et al. (2018). In a quasi-experimental validation study based on responses of 208 students (Ma et al., 2018), the SLA-LF-132 was shortened to a five-factor 73-item version, namely SLA-SF-73 which demonstrated good reliability and positive correlation with different aspects of service leadership knowledge. The five factors are (1) “Attitudes toward service and competences,” (2) “Attitudes toward moral character and caring disposition,” (3) “Attitudes toward self-reflection,” (4) Attitudes to the notion that “everyone can be a leader,” and (5) Attitudes to the notion of “implicit theory of leadership.”

In another validation study based on 2,246 undergraduate students in Hong Kong, the SLA-SF-73 was further shortened to a 46-item version (i.e., SLA-SF-46E) with seven factors via exploratory factor analyses (EFA) (Shek et al., 2018a). Seven items in the factor entitled “Implicit theory of leadership” were removed due to their low item total correlation (<0.25) (Shek et al., 2018a). Finally, the seven factors included “Vision and competence” (11-item), “People orientation” (10-item), “Caring disposition” (9-item), “Ethical role model” (5-item), “Social competence” (4-item), “Self-reflection and self-understanding” (5-item), and “Positive view about human beings” (2-item) (Shek et al., 2018a). The SLA-SF-46E is thus the latest version of Service Leadership Attitude Scale which was adopted in the present study for confirmatory factor analyses (CFA).

Although EFA is a commonly adopted method in the *initial stage* of scale development for exploring the underlying dimensional structure of a scale (Kelloway, 1995), EFA itself is not sufficient for the adequate scale validation. After the latent factor structure of a measure is identified through EFA, whether this structure is stable and applicable to other samples of data should be further validated. Researchers have commonly argued that CFA should be conducted after EFA to further validate the scale (Worthington and Whittaker, 2006). Different from EFA, CFA tests a hypothesized model based on EFA findings and other theoretical considerations (Hurley et al., 1997; Sartori and Pasini, 2007). CFA is important in scale validation because “a key validity issue is the replication of the hypothesized factor structure using a new sample” and “the most logic approach would be to conduct an EFA followed by a CFA in all cases” (Worthington and Whittaker, 2006, p. 815).

Against the above background, the major purpose of the present study was to further assess and validate the SLA-SF-46E based on a large sample of undergraduate students in Hong Kong. As only exploratory factor analyses have been conducted for SLA-SF-46E, dimensionality of the scale was further examined and validated by CFA. In addition, the study examined factorial invariance of the scale across gender and sub-groups based on “odd” and “even” case numbers of the hypothesized model. Furthermore, the study examined the reliability and convergent validity of the scale.

## Materials and Methods

### Participants and Procedure

The present sample consisted of 2,240 undergraduate students from the eight UGC-funded universities at Hong Kong (mean age = 20.44+1.64 years; 66.9% were female). We chose undergraduate students as our sample based on two major considerations. First, to nurture university students’ attitudes toward service leadership is vital for preparing them for future economy and workplace. Second, validated measures should be developed to measure Hong Kong university students’ attitudes toward service leadership because different UGC-funded universities have developed Service Leadership programs in Hong Kong. The highest proportion of the participants came from The Hong Kong Polytechnic University (20.9%), with the remaining participants evenly distributed across the other seven institutions (i.e., The Hong Kong University of Science and Technology, The Chinese University of Hong Kong, The University of Hong Kong, City University of Hong Kong, The Education University of Hong Kong, Hong Kong Baptist University, and Lingnan University). Around one third of the participants were Year 1 students (34.1%), followed by Year 2 students (26.4%), Year 3 students (19.6%), and Year 4 students (16.3%). Around 40% of the participants had taken some kind of leadership training outside of their formal university study and had known “some” to “all” knowledge about service leadership. Around 60% of the participants had taken some leadership position before participating in the present study. Table 1 presents the detailed demographic information of the participants.

**Table 1:** Demographic information.

Variables	Mean	*SD*
Age	20.44	1.639

	*n*	%

**Gender**		
Male	742	33.1
Female	1498	66.9
**Institution in which participants studied**		
The Hong Kong Polytechnic University	469	20.9
The Chinese University of Hong Kong	257	11.5
The Education University of Hong Kong	258	11.5
Hong Kong Baptist University	261	11.7
City University of Hong Kong	244	10.9
Lingnan University	251	11.2
The University of Hong Kong	253	11.3
The Hong Kong University of Science and Technology	247	11.0
**Year of commencing undergraduate study**		
2016	764	34.1
2015	591	26.4
2014	438	19.6
2013	364	16.3
2012 or before	83	3.7
**Experience of leadership training**		
Yes	969	43.3
No	1271	56.7
**Knowledge about Service Leadership (SL)**		
Have NO knowledge about SL	455	20.3
Have LITTLE knowledge about SL	812	36.3
Have SOME knowledge about SL	870	38.8
Have A LOT OF knowledge about SL	101	4.5
Have ALL the knowledge about SL	2	0.1
**Serving in leadership position**		
Yes	1348	60.2
No	892	39.8

In March 2017, students from the eight UGC-funded universities were invited to complete an online electronic survey developed by the research team. In total, 4,486 students completed the survey. Then the 4,486 participants were randomly split into two samples consisting of 2,246 and 2,240 students, respectively. While the first sample (*N* = 2,246) was used for exploratory factor analyses of the Service Leadership Attitude Scale (SLA) which was published in Shek et al. (2018a), the second sample (*N* = 2,240) was adopted in the present study for CFA of the SLA. The survey questionnaire consisted of demographic questions and a set of self-report measures, which took about 45–60 min to complete. The aim of the study and participation guidelines were clearly stated in the title page of the survey. Students were also informed that they could quit from the survey without any punishment and their personal identity and information provided would be kept strictly confidential and used solely for research purpose. Each student successfully completing the whole survey would receive a HK$100 supermarket coupon (roughly = US$12.82) as an incentive. Formal consent was obtained from the students before they started the survey.

### Measures

#### Service Leadership Attitude Scale (SLA)

The SLA adopted in this study is SLA-SF-46E, a 46-item version of SLA with seven factors (Shek et al., 2018a). As mentioned in previous sections, SLA-SF-46E is a shortened form and the latest version of SLA resulted from EFA study of the original 73-item version of SLA. Each item is rated on a six-point Likert scale (1 = “strongly disagree”; 6 = “strongly agree”). In a previous validation study, the SLA-SF-46E was reported to have good internal consistency and strong convergent validity (Ma et al., 2018; Shek et al., 2018a). As attitude to service leadership is closely related to leadership competence, character and care, we employed several measures of leadership to assess the criterion-related validity of SLA.

#### Revised Servant Leadership Profile (RSLP)

The RSLP is a 97-item scale with ten factors measuring servant leadership (Wong and Page, 2003). Service Leadership contains important elements of Servant Leadership, although it extends its scope to include serving oneself. In fact, both Service Leadership and Servant Leadership stress the meaning that leadership is a “service” and leaders need to satisfy the needs of others and of the system (Zhou et al., 2015). Besides, both models emphasize moral character, caring disposition, and self-leadership qualities of a leader (Shek et al., 2015a). They also maintain that leaders need to continuously improvement themselves in order to provide high-quality service (Shek et al., 2015a). Therefore, we included RSLP for testing the construct validity of SLA. We hypothesized that RSLP scores would be positively related to SLA scores. The participants rate all items of RSLP on a seven-point Likert scale (1 = “strongly disagree”; 7 = “strongly agree”). In the present study, five dimensions of RSLP with 20 items were utilized due to their relevance to the SLAM model of service leadership. These dimensions are “Empowering and developing others” (5-item), “Serving others” (7-item), “Open, participatory leadership” (2-item), “Inspiring leadership” (2-item), and “Courageous leadership” (4-item) (Wong and Davey, 2007, p. 5). The Cronbach’s alpha of the scale in this study was 0.95, indicating high reliability.

#### Moral Self-Concept (MSC)

According to the HKI-SLAM framework, moral character is one essential component of effective service leadership (Shek and Lin, 2015). Clearly, morality of leaders would directly influence trust-building and long-term relationship between leaders and followers or other service recipients (Shek and Lin, 2015), which would ultimately influence leadership effectiveness. As such, we hypothesized that SLA scale should be positively related to MSC. The MSC is a subscale of the Chinese Adolescent Self-Esteem Scale (CASES) developed by Cheng (1997). The CASES measures both the general and domain-specific self-concepts of adolescents in Hong Kong in seven domains, including intellectual, social, familial, moral, physical-appearance, and physical-self domains. As one subscale, the MSC aims to measure the moral dimension of self-concept of adolescents in Hong Kong. The MSC contains eight items rated on a seven-point Likert scale (1 = “strongly disagree”; 7 = “strongly agree”). One sample item is “I am a considerate person.” The Cronbach’s alpha of MSC was 0.83 in the present study, indicating good reliability.

#### Leadership Efficacy (LEF)

General leadership competence is an element of effective service leadership. Besides, an effective service leader would possess leadership efficacy (Shek and Lin, 2015). Therefore, we also adopted LEF scale in this study to test the construct validity of SLA. It was hypothesized that LEF scores would be positively related to SLA scores. The LEF is an eight-item scale aiming to measure individuals’ general leadership self-efficacy (Murphy, 1992). The participants rate the items on a five-point Likert scale (1 = “strongly disagree”; 5 = “strongly agree”). One sample item is “I am confident of my ability to influence a work group that I lead.” Good reliability as well as convergent and discriminant validities were reported in previous studies (Hoyt et al., 2003; Hoyt and Blascovich, 2010). The Cronbach’s alpha for the measure was 0.70 in this study, demonstrating acceptable reliability.

#### Interpersonal Reactivity (IRI)

In the HKI-SLAM model, service leaders should possess not only task-related but also generic competences (Shek and Lin, 2015). Among the generic competences, interpersonal competence is one important category, which helps leaders to establish and maintain positive social relationships with their followers, customers, and other people to achieve the goal of satisfying the needs of others (Shek et al., 2015). As such, we involve IRI in this study to test the construct validity of SLA. The IRI consists of 28 items with four factors, which was developed by Davis (1983) to assess empathy. In the present study, we adopted 14 items from two subscales: “Perspective taking” (7-item), and “Empathic concern” (7-item) due to their close relationship with service leadership theory. The items are rated on a five-point Likert scale (1 = “does not describe me well”; 5 = “describes me very well”). Previous study showed acceptable to good reliability of the IRI (Davis, 1980) and positive correlation between IRI and Empathy Quotient (Melchers et al., 2015) and the cognitive empathy subscale of the Brief Empathy Scale (Jolliffe and Farrington, 2006). The IRI (*a* = 0.74) demonstrated acceptable reliability in the present study.

#### Short-Form Service Leadership Knowledge Scale (SLK-SF-40)

As literature suggests a positive association between knowledge and attitude (Karki, 2014; Sung et al., 2015), we also employed SLK-SF-40 in our study to test the construct validity of SLA. The SLK-SF-40 is a 40-item scale to measure people’s mastery of important knowledge points in theory of service leadership based on multiple-choice questions (Shek et al., 2018c). Each item has a correct answer. If one participant chooses the correct answer, he/she will gain one point; otherwise, he/she will gain zero point. The SLK-SF-40 showed excellent reliability (*a* = 0.94), good structure validity, and robust convergent validity (Shek et al., 2018c). We expected that there would be a positive relationship between SLA scores and SLK-SF-40 scores.

#### Short-Form Service Leadership Behavior Scale (SLB-SF-38)

Based on the reasoned action theory, attitude could influence behavior through influencing behavioral intention (Madden et al., 1992). Empirical research also indicates that accessible and stable attitude had positive association with future behavior (Glasman and Albarracin, 2006). Therefore, we hypothesized that SLA scores would be positively associated with SLB-SF-38 scores. The SLB-SF-38 is a 38-item measure with six factors assessing people’s service leadership behavior in educational, research, and training contexts (Shek et al., 2018b). The items are rated on a six-point Likert scale (1 = “very dissimilar to me”; 6 = “very similar to me”). The SLB-SF-38 showed excellent reliability as well as structural and convergent validity (Shek et al., 2018b).

### Data Analyses

There were several steps in the CFA. First, we conducted CFA to evaluate the SLA-SF-46E, that is the latest version of the Service Leadership Attitude Scale which consists of seven factors and 46 items. Second, we tested the internal reliability of the refined scale SLA-SF-46 and its subscales by examining the related Cronbach’s alpha and mean inter-item correlation values. Third, we performed a series of measurement invariance tests to the SLA-SF-46 across gender and across two sub-samples split based on odd and even case numbers of the 2,240 sample. These tests included configural invariance test, weak factorial invariance test, strong factorial invariance test, equality of factorial covariance test, and strict factorial invariance test (Cheung and Rensvold, 2002). Configural invariance test examines whether different groups had the same conceptualization of the factor structure of the measure. Weak factorial invariance test measures whether factor loadings were the same across different groups. Strong factorial invariance test assesses whether the intercepts of items were the same for different groups. Equality of factorial covariance test checks whether factor covariance was equal across different groups. Strict factorial invariance test examines whether item residuals were the same across groups. Finally, we performed the convergent validity test by testing correlations of SLA-SF-46 with a set of leadership-related scales. Both CFA and measurement invariance tests were performed using AMOS version 23. The other tests including internal reliability and correlation tests were performed using SPSS version 25.

## Results

### Confirmatory Factor Analyses

Before performing CFA, we conducted descriptive analyses including computation of mean, standard deviation, skewness, and kurtosis of all the items. According to Curran et al. (1996), values smaller than 2 for skewness and values smaller than 7 for kurtosis can be regarded as having univariate normality of distribution. These cutoff values were adopted in the present study as criteria because they were used in different studies on CFA (e.g., Cooper et al., 2010; Perry et al., 2015). As shown in Table 2, all items demonstrated normal distribution. Therefore, we used maximum likelihood estimation for CFA and multiple indices were used to evaluate the model fit of CFA. According to Bentler and Bonett (1980), a CFI value and TLI value ≥0.90 indicate the adequate model fit. In addition, Browne and Cudeck (1993) proposed that an RMSEA value ≤0.05 indicates the “close” model fit. Findings showed that the model fit was adequate for the original seven-factor 46-item model (i.e., Model 1) of SLA (i.e., SLA-SF-46E) [χ^2^(968) = 5956.81, *p* < 0.001, CFI = 0.91, TLI = 0.90, RMSEA (90% CI) = 0.048 (0.047, 0.049), SRMR = 0.04].

**Table 2:** Descriptive statistics of the original seven-factor 46-item Service Leadership Attitude Scale (SLA-SF-46E).

Scale	Item	*M* (SD)	Skewness	Kurtosis
Subscale 1	Q14	5.12 (0.82)	−1.17	2.74
	Q15	4.99 (0.80)	−0.91	1.96
	Q20	5.02 (0.84)	−1.26	3.41
	Q21	4.96 (0.79)	−1.24	3.74
	Q22	4.90 (0.79)	−1.20	3.78
	Q23	4.80 (0.85)	−0.93	2.02
	Q24	4.98 (0.78)	−0.90	2.26
	Q26	4.99 (0.78)	−1.00	2.56
	Q27	4.94 (0.81)	−0.96	2.27
	Q28	5.08 (0.84)	−1.19	2.62
	Q33	4.86 (0.78)	−0.99	2.75
Subscale 2	Q01	4.91 (0.95)	−1.67	4.38
	Q02	4.82 (0.90)	−1.33	3.31
	Q07	4.97 (0.85)	−1.17	2.71
	Q08	5.10 (0.81)	−1.19	2.90
	Q09	4.95 (0.78)	−1.17	3.52
	Q10	5.00 (0.81)	−1.29	3.98
	Q11	5.14 (0.76)	−1.07	2.70
	Q12	4.88 (0.84)	−1.06	2.53
	Q13	4.94 (0.78)	−0.99	2.66
	Q17	4.92 (0.84)	−1.08	2.45
Subscale 3	Q52	4.80 (0.85)	−0.93	1.91
	Q53	4.72 (0.89)	−0.89	1.60
	Q54	4.66 (0.96)	−0.91	1.24
	Q55	4.60 (0.98)	−0.76	0.84
	Q56	4.64 (0.97)	−1.02	1.57
	Q57	4.76 (0.88)	−0.98	1.97
	Q58	4.79 (0.86)	−0.96	1.82
	Q59	4.76 (0.87)	−0.94	1.65
	Q60	4.86 (0.84)	−1.02	2.16
Subscale 4	Q43	4.77 (0.94)	−0.95	1.44
	Q44	4.68 (0.97)	−0.93	1.33
	Q45	4.74 (0.95)	−0.95	1.44
	Q46	4.56 (0.98)	−0.88	1.05
	Q47	4.48 (1.01)	−0.76	0.70
Subscale 5	Q35	5.15 (0.79)	−1.15	2.81
	Q37	5.11 (0.79)	−1.11	2.75
	Q38	5.07 (0.79)	−1.22	3.63
	Q41	5.04 (0.80)	−1.29	3.58
Subscale 6	Q64	4.56 (0.90)	−0.72	1.04
	Q65	4.82 (0.85)	−0.93	1.84
	Q66	5.01 (0.82)	−1.09	2.70
	Q67	4.92 (0.78)	−0.95	2.52
	Q68	5.01 (0.83)	−1.08	2.33
Subscale 7	Q03	4.33 (1.18)	−0.66	0.02
	Q04	4.31 (1.21)	−0.63	−0.15

As reported in Shek et al. (2018a), the SLA-SF-46E was adapted from the SLA-SF-73 (an earlier version of SLA consisting of 73 items) based on exploratory factor analyses (EFA). In EFA, one factor in SLA-SF-73 named “Implicit theory of leadership” (seven items) had been removed due to its low values of item-total correlation coefficients. Theoretically, “Implicit theory of leadership” refers to people’s inherent beliefs about service leadership, such as leaders are not inborn but learned and nurtured; and leaders should not over control but trust, respect, and empower their followers. These implicit values are vital in theory of service leadership (Shek et al., 2015a) and are also supported by some other leadership theories such as servant leadership (Spears, 2010) and spiritual leadership (Fairholm, 1996). As EFA is exploratory in nature and this dimension has theoretical significance, we added the seven items of this factor back to SLA-SF-46E to form an eight-factor 53-item model (i.e., Model 2). However, when we tested this hypothesized Model 2 using CFA, the model fit was only fair [χ^2^(1297) = 9339.39, *p* < 0.001, CFI = 0.87, TLI = 0.86, RMSEA (90% CI) = 0.053 (0.052, 0.054), SRMR = 0.06]. Hence, we inspected the modification indices and removed seven items which showed extreme covariance with other items within the same factor (i.e., having MI value ≥30.0), as shown in Table 3.

**Table 3:** Seven items removed from the eight-factor 53-item model of SLA due to extreme modification indices (≥30.0).

		Modification indices (MIs) with items in the same factor (≥30.0)	
Factor	Item removed	Items	Modification Indices
Factor 1: Vision and competence	Q27	Q26	133.71
		Q28	47.48
Factor 2: People orientation	Q02	Q01	527.29
		Q03	33.61
		Q08	32.02
Factor 2: People orientation	Q09	Q10	184.77
		Q08	74.37
Factor 3: Caring disposition	Q54	Q53	156.33
		Q55	71.85
		Q56	78.62
		Q58	49.28
		Q59	80.64
		Q60	50.05
Factor 3: Caring disposition	Q58	Q53	68.47
		Q54	49.28
		Q57	65.36
		Q59	74.72
Factor 8: Unchangeable and dark human nature	Q62	Q72	67.82
		Q71	103.79
		Q70	103.42
		Q63	130.48
		Q61	33.03
Factor 8: Unchangeable and dark human nature	Q72	Q71	976.61
		Q70	254.99
		Q69	33.66
		Q63	122.42
		Q62	67.82
		Q61	59.40

The refined model (i.e., Model 3) comprised 46 items with eight factors. It was then subjected to another round of CFA, which resulted in adequate model fit [χ^2^(961) = 5773.33, *p* < 0.001, CFI = 0.91, TLI = 0.90, RMSEA (90% CI) = 0.047 (0.046, 0.049), SRMR = 0.06]. Comparing Model 3 (i.e., the eight-factor 46-item model) with Model 1 (i.e., the original seven-factor 46-item model) and Model 2 (i.e., the eight-factor 53-item model), Model 3 was superior both theoretically and statistically. First, although both Model 1 and Model 3 demonstrated adequate model fit (CFI for both models = 0.91, TLI for both models = 0.90), Model 3 was more conceptually superior by incorporating the factor “Implicit theory of leadership” which represents an important theoretical dimension of service leadership attitude. Second, compared with Model 2 which showed an inadequate model fit (CFI = 0.87, TLI = 0.86), Model 3 displayed an adequate model fit (CFI = 0.91, TLI = 0.90). Besides, Model 3 was also superior to Model 1 in terms of the goodness of fit indicators. Since Model 3 and Model 1 were not nested models, we did not adopt chi-square values but Akaike information criterion (AIC) as the criteria to compare the two models. According to Burnham and Anderson (2004) and Bowen and Guo (2011), a model with a smaller AIC value should be regarded as a better-fitting model. Therefore, Model 3 (AIC = 6105.33) was regarded as superior to Model 1 (AIC = 6274.81) due to its smaller AIC value. Based on these comparisons, Model 3 (eight factors, 46 items) was finally adopted as the final version of SLA, which was named as SLA-SF-46.

**FIGURE 1:**
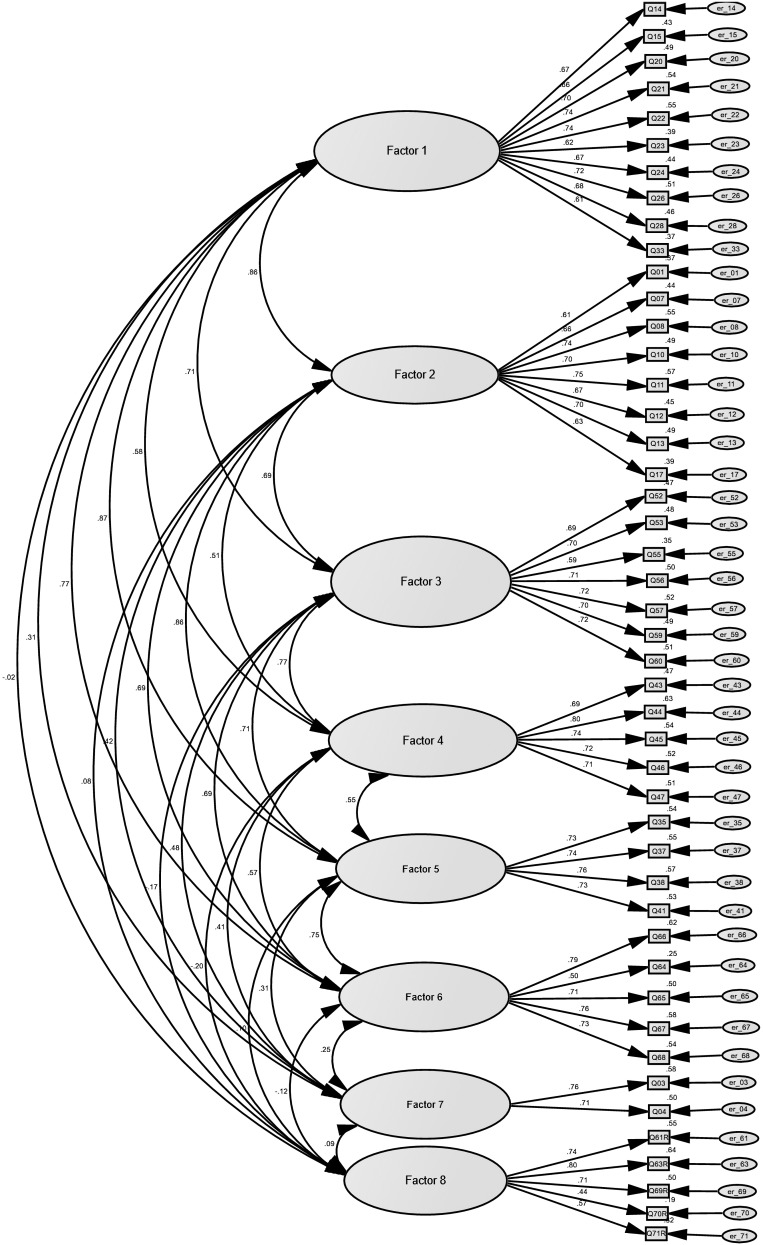
Factor model of the eight-factor 46-item SLA-SF-46.

The added factor “Implicit theory of leadership” was renamed as “Unchangeable and dark human nature” in SLA-SF-46. In short, the eight factors in SLA-SF-46 are: “Vision and competence” (10-item), “People orientation” (8-item), “Caring disposition” (7-item), “Ethical role model” (5-item), “Social competence” (4-item), “Self-reflection and self-understanding” (5-item), “Positive views about human beings” (2-item), and “Unchangeable and dark human nature” (5-item). The item standardized factor loadings in each factor are shown in Table 4. The item examples for each factor are shown in Table 5. The diagram of the factor model of SLA-SF-46 is shown in Figure 1.

**Table 4:** Standardized factor loadings for the SLA-SF-46.

Items	Factor 1	Factor 2	Factor 3	Factor 4	Factor 5	Factor 6	Factor 7	Factor 8
Q14	0.67							
Q15	0.66							
Q20	0.70							
Q21	0.74							
Q22	0.74							
Q23	0.62							
Q24	0.67							
Q26	0.72							
Q28	0.68							
Q33	0.61							
Q01		0.61						
Q07		0.66						
Q08		0.74						
Q10		0.70						
Q11		0.75						
Q12		0.67						
Q13		0.70						
Q17		0.63						
Q52			0.69					
Q53			0.70					
Q55			0.59					
Q56			0.71					
Q57			0.72					
Q59			0.70					
Q60			0.72					
Q43				0.69				
Q44				0.80				
Q45				0.74				
Q46				0.72				
Q47				0.71				
Q35					0.73			
Q37					0.74			
Q38					0.76			
Q41					0.73			
Q64						0.50		
Q65						0.71		
Q66						0.79		
Q67						0.76		
Q68						0.73		
Q03							0.76	
Q04							0.71	
Q61								0.74
Q63								0.80
Q69								0.71
Q70								0.44
Q71								0.57

**Table 5:** Item examples for each factor of SLA-SF-46.

**Factor 1: Vision and competence**
Q15. “Good leaders have the ability to make convincing arguments.”
Q21. “A good leader leads the team with inspiring and strategic vision.”
**Factor 2: People orientation**
Q1. “A good leader serves others with a genuine heart.”
Q12. “It is important for a leader to be sensitive to individuals’ specific needs.”
**Factor 3: Caring disposition**
Q55. “A good leader forgives others.”
Q56. “The ability and willingness to love followers is one of the most important leadership qualities.”
**Factor 4: Ethical role model**
Q44. “A good leader acts as an ethical model for the followers.”
Q47. “Good leaders give high priority to ethical issues.”
**Factor 5: Social competence**
Q35. “A good leader is able to collaborate with others.”
Q38. “Effective leadership largely involves good communication with followers.”
**Factor 6: Self-reflection and self-understanding**
Q67. “A leader should closely examine his/her own thoughts and behavior.”
Q68. “A true leader knows his/her own strengths and weaknesses.”
**Factor 7: Positive views about human beings**
Q3. “Everyone can be a leader regardless of his/her current role and position.”
Q4. “Everyone has the potential to be a leader.”
**Factor 8: Unchangeable and dark human nature**
Q61. “Whether or not a person can become a leader is fundamentally shaped by one’s personality and not much can be changed.” (reverse-item)
Q71. “As human beings are intrinsically lazy, a leader should closely monitor the performance of followers.” (reverse-item)

### Descriptive Statistics and Reliability

Table 6 shows the mean values, standard deviations, Cronbach’s alpha, and mean inter-item correlation for the SLA-SF-46 and its eight subscales (factors). The total scale of SLA-SF-46 and all of its eight subscales showed acceptable to excellent internal consistency (Cronbach’s alpha ranged from 0.70 to 0.93; mean inter-item correlation ranged from 0.27 to 0.55).

**Table 6:** Descriptive statistics and reliability of SLA-SF-46.

	*M*	SD	*a*	Mean inter-item correlations
SLA-SF-46 total	4.68	0.46	0.93	0.27
Factor 1: Vision and competence (10 items)	4.97	0.58	0.90	0.46
Factor 2: People orientation (8 items)	4.98	0.60	0.87	0.46
Factor 3: Caring disposition (7 items)	4.73	0.66	0.86	0.48
Factor 4: Ethical role model (5 items)	4.65	0.77	0.85	0.53
Factor 5: Social competence (4 items)	5.09	0.64	0.83	0.55
Factor 6: Self-reflection and self-understanding (5 items)	4.87	0.64	0.82	0.48
Factor 7: Positive views about human beings (2 items)	4.32	1.05	0.70	0.54
Factor 8: Unchangeable and dark human nature (5 items)	3.19	0.94	0.79	0.43

### Measurement Invariance Analyses Across Gender and Subgroups

We tested the measurement invariance of the SLA-SF-46 across gender groups. Through sequentially adding model constraints in a series of nested models, we tested configural invariance, weak factorial invariance (i.e., metric invariance), strong factorial invariance (i.e., scalar invariance), equality of factorial covariance, and strict factorial variance (Cheung and Rensvold, 2002; Millsap and Meredith, 2012). Table 7 summarizes the model fit results of different tests. The model fit for each test was acceptable. Firstly, the model fit for configural invariance test was χ^2^(1922) = 7286.28, CFI = 0.89, TLI = 0.88, RMSEA = 0.035, SRMR = 0.06. This indicates that the eight-factor structure of SLA-SF-46 was conceptualized acceptable by both gender groups. Secondly, we tested weak factorial invariance by adding the constraint of equal factor loadings to the configural invariance model. Results yielded acceptable model fit [χ^2^(1960) = 7352.50, CFI = 0.89, TLI = 0.89, RMSEA = 0.035, SRMR = 0.06]. The absolute value of ΔCFI was 0.001, supporting the weak factorial invariance across gender groups based on the criteria of ΔCFI <0.01 proposed by Cheung and Rensvold (2002).

**Table 7:** Model fit of various measurement invariance test models for gender and subgroups.

Model	*df*	χ^2^	Δχ^2^	CFI	ΔCFI	TLI	RMSEA	SRMR
**Gender**								
Configural invariance	1922	7286.28		0.89		0.88	0.035	0.06
Weak factorial invariance	1960	7352.50	66.21^∗∗^	0.89	−0.001	0.89	0.035	0.06
Strong factorial invariance	2006	7487.58	135.08^∗∗∗^	0.89	−0.002	0.89	0.035	0.07
Equality of factorial covariance	2042	7732.43	244.85^∗∗∗^	0.89	−0.004	0.88	0.035	0.10
Strict factorial invariance	2088	8528.43	796.00^∗∗∗^	0.87	−0.015	0.87	0.037	0.08
**Subgroups (groups based on “odd” and “even” case numbers)**								
Configural invariance	1922	7227.35		0.90		0.89	0.035	0.06
Weak factorial invariance	1960	7302.64	75.29^∗∗∗^	0.90	−0.001	0.89	0.035	0.06
Strong factorial invariance	2006	7353.50	50.86	0.90	−0.000	0.89	0.035	0.06
Equality of factorial covariance	2042	7433.20	79.70^∗∗∗^	0.89	−0.001	0.89	0.034	0.06
Strict factorial invariance	2088	7553.53	120.33^∗∗∗^	0.89	−0.001	0.89	0.034	0.06

*^∗∗∗^p < 0.001; ^∗∗^p < 0.01.*

In the third step, we assessed strong factorial invariance by adding the constraint of equal intercepts to the model of weak factorial invariance. The model fit was also acceptable [χ^2^(2006) = 7487.58, CFI = 0.89, TLI = 0.89, RMSEA = 0.035, SRMR = 0.07]. The absolute value of ΔCFI was 0.002, supporting the equal intercepts of the measure across gender groups. Then we tested the equality of factorial covariance across gender groups by adding a further constraint of equal factor covariance to the strong factorial invariance model. The model fit was acceptable again [χ^2^(2042) = 7732.43, CFI = 0.89, TLI = 0.88, RMSEA = 0.035, SRMR = 0.10] and the absolute value of ΔCFI was 0.004, supporting the equality of factor covariance across gender groups. Finally, we tested the strict factorial invariance by setting the measurement residues being equal on top of the equality of factorial covariance model. Results showed a marginally adequate model fit and the absolute value of ΔCFI over 0.01. Therefore, the measurement residues were not equal across male and female groups. To sum up, measurement invariance tests suggest that both male and female groups shared the same factor-structure, and had equal factor loadings, intercepts, and factor covariance regarding the SLA-SF-46.

We also tested measurement invariance of SLA-SF-46 by splitting the whole sample (*N* = 2,240) into two sub-groups based on the “odd” and “even” case numbers based on procedure adopted in research of Shek and Ma (2014) and Shek and Yu (2014). Both groups contained the same case numbers (*N* = 1,120). We conducted the same series of measurement invariance tests for the “odd” and “even” groups as we had conducted for gender groups. As shown in Table 7, all the tested models demonstrated acceptable model fit (CFI = 0.89–0.90; RMSEA = 0.034–0.035; SRMR = 0.06). The absolute values of ΔCFI for all the sequentially constrained models were smaller than the cut off value of 0.01, which indicates that the weak factorial invariance, strong factorial invariance, equality of factorial covariance, and strict factorial invariance were all well-established across “odd” and “even” groups. To sum up, results indicate that both the “odd” and “even” case-number groups shared the same eight-factor structure, equal factor loadings, intercepts, factor covariance, and measurement residues.

### Convergent Validity

To further test the validity of SLA-SF-46, correlations between the scale and its eight subscales and a set of external criterion scales measuring leadership- and inter-personal-related competences were computed. First, we predicted that service leadership attitude measure indexed by SLA-SF-46 and its subscales would be positively associated with measures of servant leadership (RSLP), moral self-concept (MSC), leadership efficacy (LEF), and Interpersonal Reactivity (IRI) because these criterion measures contain important elements of the service leadership concept proposed by HKI-SLAM. Second, as attitude is commonly related to knowledge and behavior (Glasman and Albarracin, 2006; Sung et al., 2015), we expected that SLA measures would be positively associated with service leadership knowledge and behavior measures. As shown in Tables 8, 9, there was strong support for these two sets of general expectations, suggesting that SLA-SF-46 possessed good convergent validity. For the first set of expectations, all measures of SLA were significantly related to RSLP, MSC, LEF, and IRI measures. For the second set of hypotheses, except very few exceptions, SLA measures were related to service leadership knowledge and behavior.

**Table 8:** Correlations between SLA-SF-46 and external criterion scales.

SLA-SF-46	RSLP	MSC	LEF	IRI	IRI-EC	IRI-PT
Factor 1: Vision and competence	0.48^∗∗∗^	0.49^∗∗∗^	0.19^∗∗∗^	0.36^∗∗∗^	0.30^∗∗∗^	0.35^∗∗∗^
Factor 2: People orientation	0.48^∗∗∗^	0.51^∗∗∗^	0.22^∗∗∗^	0.41^∗∗∗^	0.34^∗∗∗^	0.38^∗∗∗^
Factor 3: Caring disposition	0.52^∗∗∗^	0.44^∗∗∗^	0.19^∗∗∗^	0.33^∗∗∗^	0.25^∗∗∗^	0.33^∗∗∗^
Factor 4: Ethical role model	0.44^∗∗∗^	0.36^∗∗∗^	0.18^∗∗∗^	0.24^∗∗∗^	0.18^∗∗∗^	0.24^∗∗∗^
Factor 5: Social competence	0.48^∗∗∗^	0.50^∗∗∗^	0.19^∗∗∗^	0.42^∗∗∗^	0.35^∗∗∗^	0.39^∗∗∗^
Factor 6: Self-reflection and self-understanding	0.41^∗∗∗^	0.40^∗∗∗^	0.17^∗∗∗^	0.29^∗∗∗^	0.22^∗∗∗^	0.29^∗∗∗^
Factor 7: Positive views about human beings	0.29^∗∗∗^	0.24^∗∗∗^	0.15^∗∗∗^	0.17^∗∗∗^	0.13^∗∗∗^	0.18^∗∗∗^
Factor 8: Unchangeable and dark human nature	−0.07^∗∗∗^	0.10^∗∗∗^	0.07^∗∗∗^	0.24^∗∗∗^	0.28^∗∗∗^	0.14^∗∗∗^
SLA-SF-46 total	0.56^∗∗∗^	0.57^∗∗∗^	0.25^∗∗∗^	0.47^∗∗∗^	0.39^∗∗∗^	0.43^∗∗∗^

*^∗∗∗^p < 0.001; SLA-SF-46, eight-factor, 46-item Short Form Service Leadership Attitude Scale; RSLP, Revised Servant Leadership Profile; MSC, Moral Self-Concept; LEF, Leadership Efficacy; IRI, Interpersonal Reactivity Index; IRI-EC, Empathic Concern subscale of IRI; IRI-PT, Perspective Taking subscale of IRI.*

**Table 9:** Correlations between SLA-SF-46 and other service leadership scales and subscales.

SLA-SF-46	SLK-SF-40	SLB-SF-38 total	SLB-SF-38 Factor 1	SLB-SF-38 Factor 2	SLB-SF-38 Factor 3	SLB-SF-38 Factor 4	SLB-SF-38 Factor 5	SLB-SF-38 Factor 6
Factor 1	0.31^∗∗∗^	0.51^∗∗∗^	0.46^∗∗∗^	0.52^∗∗∗^	0.35^∗∗∗^	0.42^∗∗∗^	0.38^∗∗∗^	0.26^∗∗∗^
Factor 2	0.34^∗∗∗^	0.49^∗∗∗^	0.42^∗∗∗^	0.55^∗∗∗^	0.33^∗∗∗^	0.43^∗∗∗^	0.34^∗∗∗^	0.26^∗∗∗^
Factor 3	0.14^∗∗∗^	0.51^∗∗∗^	0.42^∗∗∗^	0.52^∗∗∗^	0.37^∗∗∗^	0.41^∗∗∗^	0.34^∗∗∗^	0.40^∗∗∗^
Factor 4	0.07^∗∗^	0.40^∗∗∗^	0.33^∗∗∗^	0.41^∗∗∗^	0.29^∗∗∗^	0.31^∗∗∗^	0.26^∗∗∗^	0.35^∗∗∗^
Factor 5	0.38^∗∗∗^	0.50^∗∗∗^	0.44^∗∗∗^	0.56^∗∗∗^	0.32^∗∗∗^	0.45^∗∗∗^	0.33^∗∗∗^	0.22^∗∗∗^
Factor 6	0.23^∗∗∗^	0.47^∗∗∗^	0.43^∗∗∗^	0.47^∗∗∗^	0.34^∗∗∗^	0.38^∗∗∗^	0.36^∗∗∗^	0.22^∗∗∗^
Factor 7	0.03	0.27^∗∗∗^	0.20^∗∗∗^	0.28^∗∗∗^	0.23^∗∗∗^	0.23^∗∗∗^	0.13^∗∗∗^	0.27^∗∗∗^
Factor 8	0.40^∗∗∗^	−0.05^∗^	−0.03	0.02	−0.09^∗∗∗^	0.02	−0.05^∗^	−0.18^∗∗∗^
SLA-SF-46 total	0.38^∗∗∗^	0.58^∗∗∗^	0.50^∗∗∗^	0.62^∗∗∗^	0.39^∗∗∗^	0.49^∗∗∗^	0.40^∗∗∗^	0.33^∗∗∗^

*^∗∗∗^p < 0.001; ^∗∗^p < 0.01; ^∗^p < 0.05; SLA-SF-46, eight-factor, 46-item Short Form Service Leadership Attitude Scale; SLK-SF-40, The 40-item short version Service Leadership Knowledge Scale; SLB-SF-38, The 38-item short version Service Leadership Behavior Scale.*

## Discussion

This study attempted to validate the Service Leadership Attitude Scale (SLA) in Hong Kong which assesses different aspects of a person’s attitude toward service leadership. The construct was developed based on envisioning the importance of service leadership in organizational success in contemporary service-based economy and in developing university students’ attitude toward service leadership. The construct was also developed in response to a lack of validated measures to assess university students’ service leadership attitude. The SLA construct was developed based on the service leadership framework promoted by HKI-SLAM (Shek and Lin, 2015) and a careful examination of important leadership concepts in the leadership literature (Ma et al., 2018). It incorporated several key aspects of leadership development that are perceived as important in service-based economy such as distributed leadership, self-leadership, and competence, character and caring dimensions of leadership. To understand the psychometric properties of the developed measure, we conducted different analyses to understand the reliability and validity (factorial validity and convergent validity) of the scale.

The present study has two major strengths. One was its large sample size. According to Kyriazos (2018), “the factor pattern developed by a large-scale factor analysis is probably more stable than that based on a small sample size” (p. 2208). As such, the sample size of 2,240 in this study with participants coming from different study years and universities of Hong Kong would make the validation results more stable. Another strength of this study is employment of CFA to understand the factorial invariance of the final model in assessing the factorial validity of the measure.

The purpose of CFA was to yield a factor structure of SLA not only statistically satisfactory but also theoretically sound (Hurley et al., 1997). In previous exploratory factor analyses (Shek et al., 2018a), one factor dimension named “Implicit leadership theory” with seven items was removed due to their low item-total correlations. This dimension measures individuals’ underlying beliefs regarding whether leadership can be changed or not and whether leader-follower relationship is a strict-control or mutual-trust one. To strengthen the soundness of the theoretical dimensions of the construct, we added the dimension “Implicit leadership theory” back to the existing seven-factor 46-item SLA-SF-46E to further examine and refine the factor structure of the construct by using CFA. Since model fit indices of the newly formed eight-factor 53-item SLA showed unsatisfactory model fit, we removed seven items showing large modification indices with other items in the same factor because large modification indices possibly indicate that these items had highly similar contents with other items in the same factor (Bray and Harvey, 1992). Similar examples of refining CFA models based on modification indices are common in the literature on scale development (e.g., Benson and Bandalos, 1992; Artino et al., 2010; Ng et al., 2011). As expected, the refined eight-factor 46-item model of SLA (i.e., SLA-SF-46) showed an adequate and better model fit than the seven-factor 46-item model and eight-factor 53-item model. The CFA results also indicate the stability of different theoretical dimensions of service leadership attitude, which further proves the soundness of the theoretical framework of service leadership proposed by HKI-SLAM (Chung, 2011; Shek and Lin, 2015) and in different literature on service leadership (Testa, 2004; Gronfeldt and Strother, 2006; Snell et al., 2015a).

The measurement invariance tests attempted to determine if the SLA-SF-46 measures the same construct for participants from different groups (Cheung and Rensvold, 2002) through comparing a series of increasingly constrained CFA models (Schoot et al., 2012). The existence of configural invariance indicates that participants from both genders and from both “odd” and “even” case number groups had the same conceptualization of the construct (i.e., they attributed the same subset of items to the same factor dimension). While different groups might agree with the factor structure and what items belong to what factor, they may not agree with the factor loading for each item. Therefore, we tested weak factorial invariance, the existence of which assured that the factor loading parameters of all items were equal across different groups. The fulfillment of strong factorial invariance meant that item intercepts were also invariant across groups. The covariance among factors was also equal across different groups. Finally, we tested whether the item residuals (i.e., the measurement error that each item measures the latent construct) were equal across groups to examine the strict factorial invariance. While the residuals were equal across “odd” and “even” case number groups, they were not equal across gender groups. According to different scholars such as Millsap and Meredith (2004) and Schoot et al. (2015), the strict factorial invariance actually could hardly be met even based on large samples. Cheung and Rensvold (2002) argued that residual variance might be caused by many different reasons such as “difference in vocabulary, idioms, grammar, syntax, and the common experiences of different cultures” (p. 237). Some scholars suggested that the test for strong factorial invariance is sufficient for assessing measurement invariance (Stoel et al., 2004). Considering suggestions in the literature, it could be argued that the SLA-SF-46 construct is equal across gender groups and across “odd” and “even” case groups. The measurement invariance tests further strengthened the validity and stability of the factor structure and theoretical dimensions of the scale in measuring attitude toward service leadership.

Testing convergent validity is one of approaches to establish the validity of a construct by demonstrating whether and to what extent a measure is correlated with other theoretically related measures (i.e., they assess similar information) (Carlson and Herdman, 2012). To test the convergent validity of SLA-SF-46, we employed several established measures such as RSLP, MSC, LEF, and IRI because these measures assess constructs closely related to service leadership. Since the service leadership concept incorporates important elements of servant leadership such as focus on leaders’ moral character, caring disposition, and visioning attributes (Wong and Page, 2003), the significant correlation between the two constructs indicates that SLA-SF-46 could measure attitudes toward these important dimensions of service leadership. In addition, because MSC, LEF, and IRI measure moral self-concept (Cheng, 1997), leadership self-efficacy (Murphy, 1992), and empathy (Davis, 1983), respectively, which are closely related to service leadership (Shek and Lin, 2015), the significant associations of SLA-SF-46 with these constructs indicate that SLA-SF-46 is a valid measure in assessing individuals’ attitude toward these different dimensions of service leadership. Furthermore, as the literature suggests that attitude could be shaped by knowledge and could shape behavior through influencing behavioral intention (Ajzen, 1991; Anker et al., 2010), the significant association between SLA-SF-46 and the newly validated Service Leadership Knowledge Scale and Service Leadership Behavior Scale further adds to the evidence that SLA-SF-46 is a valid measure to assess attitudes toward service leadership.

To measure the reliability of a construct, internal consistency analyses using Cronbach’s alpha were conducted (Heale and Twycross, 2015). Reliability analyses of the total scale and the subscales revealed that the different measures had good internal consistency as reflected by the values of coefficient alpha and mean inter-item correlation coefficients. This indicates that the items within different factor dimensions are measuring the same factors, which was the prerequisite for establishing the validity of the construct (Kimberlin and Winterstein, 2008).

Although the present study is pioneering and the findings are robust, there are several limitations. First, although the sample was large and the participants came from different higher education institutions of Hong Kong, the sampling method was not random in nature. Hence, it would be helpful to collect random samples of university students in Hong Kong. Second, as this study was the first scientific work conducted to validate the SLA via CFA at Hong Kong, more validation work should be done to replicate the present findings in other adolescent and adult samples. Third, while the reliability of the construct has been tested through assessing the internal consistency of the measure, other types of reliability test such as the test-retest reliability should be conducted in future research to verify the time stability of the construct (Heale and Twycross, 2015). Finally, it would be helpful to examine how service leadership attitude may predict service leadership behavior over time (i.e., predictive validity). Despite the limitations, the present study contributes significantly to the development of valid measures of attitudes to service leadership in Hong Kong.

## Ethics Statement

The study was approved by the Human Subjects Ethics Sub-committee (HSESC) (or its Delegate) of The Hong Kong Polytechnic University. All subjects have given written informed consent before start of the study.

## Author Contributions

DS designed the research project and contributed to all steps of the project and the manuscript. WC contributed to the data interpretation of the study, prepared the initial draft of the manuscript, and revised the manuscript based on the comments and revisions provided by DS.

## Conflict of Interest Statement

The authors declare that the research was conducted in the absence of any commercial or financial relationships that could be construed as a potential conflict of interest.
